# The impact of strategic alliances on corporate real investment and its mechanisms from the perspective of organizational learning theory: An empirical analysis based on Chinese listed companies

**DOI:** 10.1371/journal.pone.0332364

**Published:** 2025-09-19

**Authors:** Kai Gao, Xinlei Huang

**Affiliations:** 1 Antai College of Economics and Management, Shanghai Jiao Tong University, Shanghai, China; 2 School of Management, Shanghai University of Engineering Science, Shanghai, Huadong, China; Fooyin University, TAIWAN

## Abstract

This study examines how firms enhance resource allocation efficiency and stimulate real investment through strategic alliances. The findings, based on data from Chinese A-share listed companies on the Shanghai and Shenzhen Stock Exchanges from 2009 to 2022, indicate a significant positive correlation between strategic alliances and corporate real investment. This suggests that strategic alliances effectively improve resource allocation efficiency and promote investment in the real economy. By differentiating between equity-based and bilateral contractual strategic alliances, the results highlight a significantly positive impact of equity-based strategic alliances on corporate real investment. Path analyses further reveal that strategic alliances promote real investment growth by stimulating innovation momentum, enhancing risk-taking capacity, and strengthening innovation capability. Additional analyses indicate that companies with robust capital acquisition abilities—characterized by high commercial credit, patient capital, and government subsidies—can significantly enhance the positive effect of strategic alliances on real investment. This study enriches theoretical discourse on the economic consequences of strategic alliances, and offers policy recommendations for firms to optimize resource allocation and for governments to effectively guide capital toward the real economy.

## 1. Introduction

Strategic alliances are cooperative arrangements in which multiple enterprises share property rights and strategic decision-making authority to achieve objectives such as resource sharing and enhanced competitiveness, while maintaining independent ownership and control over other assets [1, 2]. With the announcement of new tariff policies by the Trump administration in the United States, the global economic environment has become increasingly tense, and enterprises are facing intensified competitive pressures. To mitigate risks associated with fluctuations in the external environment [3], a growing number of enterprises are engaging in strategic alliances to share resources, enhance innovation capabilities, reduce production costs, and thereby expand their market share. Additionally, economic globalization and rapid technological advancement are intensifying market competition, prompting enterprises to leverage strategic alliances to expand their sales networks, acquire technological coordination, and lower research and development (R&D) costs, ultimately enhancing enterprise value and competitiveness in global markets [4]. From the perspective of organizational learning theory, strategic alliances are not only platforms for resource sharing but also important environments for collaborative learning. In strategic alliances, firms engage in dynamic learning and knowledge accumulation through the sharing of knowledge, technology, and experience with their partners. This learning process helps firms enhance their resource integration capabilities, optimize decision-making mechanisms, and achieve more efficient resource allocation.

Current research on strategic alliances has successively analyzed the market reactions to strategic cooperation announcements [5], spillover effects of strategic alliances [6], and the impact of participation in strategic alliances on corporate activities [7], corporate governance, financing costs, corporate performance, corporate governance structure, earnings management [8], research investment [9], and innovation output [10]. These studies have identified the positive effects of strategic alliances in enhancing corporate value, improving corporate governance, and promoting R&D innovation, providing empirical evidence for the value effects generated by strategic alliances from various perspectives. However, research has generally focused on analyzing how strategic alliances create short-term gains or drive innovation activities through the integration of external resources and the promotion of synergies. There is less research on how alliances optimize resource allocation within firms and improve corporate performance. To date, there is no economic analysis of the resource allocation consequences of strategic alliances, no literature has tested the impact of strategic alliances on corporate entity investments, nor has the mechanism through which strategic alliances affect corporate entity investments been explained, let alone any empirical evidence provided.

Strategic alliances offer collaborative advantages such as resource sharing and transaction cost reduction. However, whether and how strategic alliances influence firms’ long-term investment behavior through the development of organizational capabilities remains underexplored. This study adopts the perspective of organizational learning, positing that strategic alliances are not merely platforms for resource allocation, but also critical mechanisms through which firms absorb knowledge, accumulate capabilities, and update cognitive frameworks. Through continuous interaction and cooperation within alliances, firms can enhance innovation capacity, improve resource integration, and strengthen risk-bearing ability, thereby enabling more effective real investment in areas such as R&D and market expansion. This perspective redefines the function of strategic alliances, shifting from static resource acquisition to dynamic learning processes. It contributes to a better understanding of how strategic alliances promote real investment and enriches the theoretical discourse on alliance-based corporate development.The purpose of this study is to explore how firms’ participation in strategic alliances affects their corporate investments from the perspective of organizational learning theory. Specifically, this study aims to answer the following two questions: (1) How does a firm’s participation in strategic alliances impact its corporate investments? (2) What capabilities of firms can accelerate the positive effect of strategic alliances on corporate investments?

The findings show that strategic alliances significantly promote corporate real investment. Specifically, strategic alliances enhance firms’ risk-taking capacity, reduce managerial short-termism, and strengthen corporate innovation capabilities, thereby positively influencing corporate real investment. These conclusions stand up to rigorous robustness and endogeneity tests. Further analysis reveals that strategic alliances have a stronger positive impact on corporate real investment among enterprises with greater capital acquisition capabilities.

The contributions of this paper are as follows: Theoretical significance, it identifies the positive impact of strategic alliances on corporate real investment, thereby deepening understanding of the economic consequences of strategic alliances and extending this literature to the domain of real investment. Existing research primarily concentrates on economic outcomes under external conditions surrounding alliance formation [11], with limited attention given to resource allocation efficiency, particularly in relation to real investment. Thus, this study enhances understanding of the strategic benefits of alliances in terms of aiding firms in optimizing resource allocation, strengthening innovation capabilities, expanding market channels, and reinforcing competitive advantages. Second, the study enriches the literature on factors influencing corporate real investment, clarifying the internal mechanisms through which strategic alliances foster real investment, as well as the external capital acquisition mechanisms involved. Prior studies on corporate real investment predominantly examine either macro-environmental or firm-level microeconomic factors [12,13]. Empirical significance This study constructs an analytical framework incorporating mediating mechanisms—including innovation capability, managerial short-termism, and risk-bearing capacity—as well as moderating mechanisms such as commercial credit, patient capital, and government subsidies. It systematically identifies the multiple pathways and boundary conditions through which strategic alliances influence corporate real investment. Unlike traditional research that primarily focuses on macro-level policies or firm-specific characteristics affecting investment behavior, this paper is among the first to embed strategic alliances within the context of corporate resource allocation. It empirically verifies the critical role of alliances in promoting long-term investment and provides methodological and data-based support for future related studies. Practical significance, this study provides valuable insights for regulatory authorities and corporate management regarding the improvement of resource allocation capabilities. The findings suggest that strategic alliances, as increasingly prevalent corporate strategies, positively affect real investment, and that stronger capital acquisition capabilities significantly amplify this effect. Consequently, this paper recommends that regulatory bodies and management proactively engage financial institutions and other investors through strategic alliances to promote the realization of long-term investment projects, which holds important implications for enterprise growth and the mitigation of systemic financial risks.

## 2. Literature review

### 2.1. Research on strategic alliances

The concept of strategic alliances was actively promoted and implemented in practice by Roger Nagel of the Digital Equipment Corporation (DEC) in the United States; nevertheless, the idea did not achieve widespread recognition during its initial development [14,15]; Currently, there is no universally accepted definition of strategic alliances, as they represent a dynamically evolving concept. Motivated by rationality considerations, strategic alliances constitute a production organization model whereby firms engage in specialization and division of labor to reduce transaction costs and achieve efficient production [16]. Strategic alliances may take diverse forms, including joint ventures, cooperative R&D activities, production agreements, marketing or distribution agreements, and technology transfers [17]. Generally, strategic alliances are defined as short-term or long-term cooperative partnerships formed between two or more enterprises pursuing mutual goals. Essentially, such alliances involve exchange, sharing, and joint development, enabling partner firms to complement each other’s advantages and share risks [18,19]. Strategic alliances can be either equity-based or contractual (non-equity-based) [19]. Equity-based alliances often involve mutual equity holdings or joint ventures, whereas contractual alliances typically represent looser collaborative agreements without the establishment of independent economic entities. Additionally, alliances can be categorized as R&D-based or non-R&D-based. From a resource perspective, strategic alliances represent organizational arrangements whereby enterprises expand their boundaries, exchange information, and share resources to achieve strategic objectives, thereby deepening the value of alliances from a long-term perspective [20]. Through equity participation or contractual methods, firms leverage complementary advantages to share resources and technology, distribute risks, and jointly obtain benefits [21]. As a novel form of economic organization situated between markets and hierarchical organizations, strategic alliances help avoid the drawbacks inherent in purely market-based or internal governance arrangements, thereby reducing transaction costs [22] and enhancing strategic resource acquisition [23]. Strategic alliances can be further conceptualized as networked organizations, which comprise two or more firms that leverage their respective strategic resources to achieve common strategic objectives and interests, share risks, and enhance their organizational learning efficiency [24]. Thus, strategic alliances have become an essential platform for firms to create value by effectively identifying, absorbing, and internalizing complementary and heterogeneous resources and knowledge from alliance partners [25,26].

Regarding the economic consequences of strategic alliances, scholars widely recognize their positive role in enhancing firm performance and innovation, which ultimately improves enterprise value. With respect to firm performance, first, alliances characterized by functional diversity and high-quality partners significantly enhance firm performance [27]; however, for certain types of entrepreneurial firms, maintaining excessive alliances may generate negative effects [28]. This indicates that optimizing the alliance portfolio in terms of size and partner quality is crucial for performance improvement [29]. Second, merely entering strategic alliances does not automatically guarantee improved performance; organizational behaviors serve as critical intermediary factors. Through enhanced communication, increased trust, and accumulated relational capital, enterprises can effectively transform alliance resources into tangible benefits while reducing transaction costs and dispersing operational risks [30,31,32]. Third, the level of fairness perception within alliance networks determines the effects of alliances on performance. Higher perceived fairness positively moderates the relationship between alliance scale and corporate performance [33]. In terms of innovation, strategic alliances significantly enhance corporate innovation capabilities through knowledge sharing, the promotion of knowledge flow, governance optimization, and resource integration [34]. Furthermore, contractual governance within alliances provides clear division of responsibilities and rules for benefit allocation, effectively mitigating risks such as opportunism and conflicts of interest [35,36].

### 2.2. Research on corporate real investment

Corporate real investment is a critical means through which enterprises achieve effective resource allocation. In recent years, the question of how to promote a shift from financialization to real economic activities has gained increasing scholarly and policy attention. Existing literature generally explores the influencing factors of corporate real investment from the perspective of either the external macroeconomic environment or the internal micro-level firm characteristics. From the macroeconomic perspective, various factors have been found to impact corporate real investment. These include monetary policy [37], the level of environmental information disclosure [38], China’s Belt and Road Initiative [39], economic policy uncertainty [40], and the VAT reform policy [41,42]Each of these elements can either constrain or stimulate investment in the real economy by altering financing conditions, expectations, and firm-level strategic responses.

From the micro-level perspective, the internal characteristics and strategic choices of firms—particularly digital transformation—play a crucial role in enhancing real investment. Digitalization has been shown to significantly optimize resource allocation and corporate governance structures [12], thereby improving both the efficiency and confidence in real investment. On the one hand, the performance, breadth, and depth of digitalization facilitate resource integration and utilization, reduce investment risks, and improve investment efficiency, enabling firms to focus more effectively on core business development. On the other hand, the integration of digital technologies with traditional business models fosters structural adjustment and innovation in production methods, organizational management, and financial oversight, ultimately leading to enhanced returns on real asset investments [13]. Moreover, there is a documented negative correlation between financial investment and real investment [43], suggesting a crowding-out effect. As emerging tools for corporate supervision, digital technologies help optimize governance structures [12], further strengthening firms’ confidence in real investment. This digital-driven shift supports the return of manufacturing firms to the real economy and contributes to their pursuit of high-quality development.

## 3. Theoretical framework and research hypotheses

### 3.1. Strategic alliances and corporate real investment

According to organizational learning theory, strategic alliances serve as a vital mechanism through which firms acquire, absorb, and internalize diverse and heterogeneous resources and knowledge, forming a foundation for sustained value creation. While existing literature has expanded the study of strategic alliances from various theoretical perspectives—including resource dependence theory, transaction cost economics, and the knowledge-based view—there is a general consensus that alliances play a significant role in enhancing organizational learning and knowledge acquisition. based on the core framework of organizational learning theory and existing research findings, proposes that corporate participation in strategic alliances generally contributes to enhancing the level of their real investment [44,45].

First, Strategic alliances enhance corporate innovation capability through knowledge acquisition and absorption, thereby driving corporate real investment. Strategic alliances provide firms with effective channels for acquiring new knowledge. By interacting with partners, firms can access external resources such as technology, market insights, and management experience. Through mechanisms such as knowledge acquisition, integration, and absorption, firms continuously accumulate and create new knowledge, which in turn drives the enhancement of their innovation capability [46]. Firms can foster innovation through interactions with alliance partners by transforming both explicit and tacit knowledge. Moreover, a firm’s innovation capability is not only dependent on its internal R&D capabilities but also heavily relies on its ability to absorb external knowledge [47]. By collaborating with different types of partners and restructuring new and existing alliances, firms can expand their knowledge boundaries, internalizing individual and shared knowledge from the alliance network into proprietary resources. This enhances their resource allocation capacity, particularly in innovation and other domains [48]. Active cooperation with other entities within the alliance effectively reduces the inherent risks of pursuing innovation in uncertain environments. This process of acquiring and absorbing new knowledge through collaboration not only enhances the firm’s innovation capability but also optimizes the efficiency of resource allocation. By participating in collaborative innovation platforms such as R&D alliances and industry technology alliances, firms can integrate internal and external resources, optimize core knowledge reserves, and enhance their ability to absorb and reapply external knowledge. This process significantly improves both the quantity and quality of innovation outputs, allowing firms to reduce production costs and improve product quality during the technological innovation process. Consequently, it provides a solid economic foundation for future corporate real investment [49]. The improvement in innovation capability drives firms to focus more on long-term real investments, optimizing investment structures and reducing short-term speculative behavior. After enhancing their technological advantages and absorptive capacity through innovation, firms can narrow the technological gap, broaden knowledge acquisition channels, and strengthen their competitive advantage. By achieving more efficient resource allocation, firms can maintain longer-lasting competitiveness [50]. In conclusion, strategic alliances provide firms with diversified channels for knowledge acquisition, help enhance innovation capability, and further encourage increased investment in long-term real assets such as R&D facilities, production equipment, and technological platforms. This leads to the transformation of knowledge into innovation, ultimately increasing corporate real investment.

Second, strategic alliances enhance a firm’s risk-bearing capacity through knowledge sharing and risk perception, thereby driving corporate real investment. By promoting information sharing and experience exchange between firms, strategic alliances increase firms’ understanding of environmental uncertainties, enhancing their ability to identify and respond to risks. Through cooperation with alliance partners, firms not only gain more technological and market resources but also absorb experiential knowledge in risk management. This knowledge-sharing process significantly improves managers’ risk perception and decision-making confidence, enabling firms to more comprehensively identify project risks and take effective measures to address challenges. As the organization’s risk response capabilities improve, firms gradually develop a greater willingness to bear risk, and become more inclined to invest in high-risk, high-return long-term real projects such as fixed assets, technological upgrades, and industry upgrades [51]. The long-term cooperative relationships and mutually beneficial cooperation models in strategic alliances allow firms to establish stable partnerships, ensuring long-term stable profit streams and operational performance [52], thus increasing their risk-bearing capacity. Furthermore, the coordination of procurement, marketing, and distribution processes within the alliance can effectively reduce operational and financing costs, alleviating the financial constraints faced by the firm [53]. As the alliance network continues to grow, firms can not only learn crisis management experiences from their partners to fill their own knowledge gaps but also internalize these lessons, further enhancing their risk identification, assessment, and response capabilities. This greatly promotes the firm’s risk perception and learning ability [54]. In long-term strategic alliance cooperation, member firms become interdependent, adhering to the principle of “harmful to others, harmful to oneself,” forming an implicit governance mechanism. This mechanism, through mutual supervision and self-restraint, mitigates the occurrence of moral hazards and adverse selection. The synergistic effect of this mechanism enhances the firm’s resource integration capabilities, risk-avoidance ability, and organizational resilience, significantly improving overall risk-bearing capacity [55]. Higher risk-bearing capacity is often accompanied by a more optimized capital structure and lower financing costs, enabling firms to allocate more resources to long-term investment projects. Ultimately, firms with strong risk-bearing capacity can manage capital more effectively, reduce external financing costs, and ensure that corporate real investments receive adequate funding support. This allows firms to mitigate the impact of external shocks and market fluctuations and strengthens confidence in long-term investments. In conclusion, strategic alliances enhance firms’ risk-bearing capacity through knowledge sharing and risk perception learning, driving more strategic real investments. This, in turn, improves resource allocation efficiency and strengthens long-term competitiveness.

Finally, strategic alliances mitigate managerial short-termism through double-loop learning, thereby driving corporate real investment. Strategic alliances encourage management to reflect on the alignment between short-term goals and the long-term interests of the company, helping to curb managerial tendencies toward short-termism. Compared to ordinary business collaborations, strategic alliance members are more reliant on a long-term, stable foundation of mutual trust. This trust-based relationship is not only the core governance mechanism for the smooth operation of the alliance but also an important safeguard for maintaining long-term cooperation between member firms [56]. The trust and close ties within the alliance prompt managers to prioritize the collective interests of the alliance and the long-term development goals of the firm, thus alleviating the short-term incentives caused by agency problems, broadening managers’ decision-making horizons, and reducing the tendency to overly rely on short-term financial returns [57]. Double-loop learning, as an important component of organizational learning theory, encourages firm managers to reevaluate and adjust their existing strategic assumptions and values, making them more likely to focus on the company’s long-term strategic goals and sustainable value growth, rather than short-term performance. This cognitive shift drives the firm’s resources away from short-term projects and toward long-term capital expenditures, such as investments in fixed assets, long-term R&D facilities, and production expansion projects, which significantly enhances the firm’s willingness to make real investments. At the same time, ongoing media attention, as an external governance mechanism, strengthens the necessity of managing the firm’s public image and enhances the supervision of managerial behavior. This external pressure urges managers to act more cautiously, curbing short-term opportunistic behaviors and minimizing reputational risks. With a more cautious approach to governance matters, managers reduce the impact of short-term actions on decision-making, ensuring strategic consistency and avoiding the negative effects of frequent resource reallocation and short-term performance fluctuations. Short-sighted managers often use their control over resources and power to influence the scale and direction of investments, pursuing short-term goals that may disrupt the firm’s strategic direction and hinder the implementation of long-term investment plans. Through the double-loop learning mechanism of strategic alliances, firms can effectively mitigate this short-termism tendency, ensuring the coherence of their strategic direction, thereby achieving long-term, sustained growth in real investments and improving overall profitability [58].

Based on the above analysis, the following hypotheses are proposed:

**Hypothesis 1:** Participation in strategic alliances enhances corporate real investment by improving the efficiency of resource allocation.
  **Hypothesis 1a:** The stimulation of corporate innovation capability serves as a mediating mechanism through which strategic alliances influence real investment.
  **Hypothesis 1b:** The enhancement of corporate risk-bearing capacity serves as a mediating mechanism through which strategic alliances influence real investment.
  **Hypothesis 1c:** The reduction of managerial short-termism serves as a mediating mechanism through which strategic alliances influence real investment.

### 3.2. The moderating role of capital acquisition capability

Building upon the foundation of strategic alliances, capital acquisition capability plays a crucial role in firms’ resource allocation processes. It serves not only as the core financial safeguard but also as a fundamental prerequisite for the smooth implementation of real investment decisions. Given the capital-intensive, long-term, and delayed-return nature of real investment, enterprises face elevated demands for sustained and reliable capital support. If firms are constrained by limited financing channels and weak capital acquisition capacity, their efficiency in resource allocation will be directly impaired, resulting in investment delays or cancellations and weakening the strategic alliance’s capacity to facilitate the shift from financialization to real economic activities. Meanwhile, institutional endorsement, policy support, and a firm’s own financial health—key contingency factors in the external environment—profoundly influence strategic decision-making and corporate responsiveness. This study uses capital acquisition capability as a proxy for external environmental conditions, with the aim of revealing how this moderates the value-realization pathways of strategic alliances. Furthermore, it seeks to identify the boundary conditions under which strategic alliances affect corporate real investment across varying capital environments, thereby offering a more comprehensive understanding of the generalizability and applicability of alliance effects.

*The Moderating Role of Patient Capital*. Patient capital is typically defined as a form of equity or debt investment characterized by a long-term horizon, diverse sources of funding, and low liquidity requirements [59]. This form of capital emphasizes the co-creation of value and sustainable development, making it particularly suitable for alliance-based cooperation that requires ongoing investment—such as breakthrough innovation or long-term infrastructure projects [60]. Within strategic alliances, partner firms collaborate through resource sharing, co-innovation, and joint governance to pursue mutual growth. However, such collaboration often faces constraints related to extended investment cycles and uncertain returns. This is especially true for large-scale projects where financial stability and durability are paramount.

The introduction of patient capital effectively alleviates the funding constraints faced by alliance members in pursuing medium- to long-term projects. It provides sustained financial support for resource coordination and cooperative activities within the alliance, while simultaneously enhancing firms’ capacity to withstand risk and uncertainty. This, in turn, incentivizes managerial efforts to pursue long-term investments within the framework of the alliance [61]. Under these conditions, the inherent advantages of strategic alliances in terms of information sharing, resource integration, and risk sharing are more fully realized. The support of patient capital amplifies the synergistic effects of alliances, enabling partner firms to translate cooperative potential into tangible, real investment behaviors such as equipment upgrades, capacity expansion, and process optimization—investments typically characterized by longer payback periods. These capital-intensive yet strategically aligned investments contribute to stable returns and help strategic alliances generate more substantial and sustained impact in promoting the transition from virtual to real economic activity [62].

*The Moderating Role of Government Subsidies.* Government subsidies, as a crucial policy instrument for correcting market failures, play a pivotal role in reinforcing the positive effect of strategic alliances on corporate real investment. By directly increasing non-operating income, subsidies enhance a firm’s tolerance for innovation failure and investment uncertainty, thereby indirectly boosting managerial confidence to engage in long-term projects and real investments within strategic alliances. In alliance contexts, firms often face capital-intensive and high-risk collaborative tasks, such as joint R&D and capacity expansion. Targeted government subsidies help alleviate financial pressure, and reduce internal resistance and hesitation, among alliance members regarding real investment. Subsidies also carry a significant signaling effect. When firms receive substantial government support within the framework of a strategic alliance, external investors interpret this as a positive endorsement of the firm’s strategy and the broader industry’s development potential. This boosts market confidence, eases financing constraints, and opens up additional funding channels for real investment within the alliance [63,64]. Furthermore, firms can use subsidy funds to hedge against external risks such as currency fluctuations, thereby enhancing profitability and building a stronger financial foundation for future investments. Empirical studies have shown that government subsidies significantly increase firms’ innovation output [65,66]. As the level of public support intensifies, the synergistic effects of alliances are amplified. Firms become more proactive in integrating resources, sharing technologies, and focusing on core operations. This increases innovation incentives and enables strategic alliances to more effectively serve the “virtual-to-real” transition goals of enterprises [67].

*The Moderating Role of Commercial Credit.* Strategic alliances are often built on supply chain cooperation, resource sharing, and joint development. Through frequent transactions and trust accumulation, firms strengthen cooperative stickiness and create favorable conditions for building commercial credit. Prior studies examine the influence of both internal and external factors on commercial credit dynamics [68–71] As a financial “bridge” within supply chain transactions, commercial credit plays a key role in alleviating funding pressures faced by firms engaged in real investment under alliance frameworks—particularly in contexts where bank financing is constrained and capital market volatility is high. From the perspective of the competitive hypothesis of trade credit, stronger and more complementary alliances lead to improved bargaining power and reputation within the industry, enabling firms to develop tighter, more resilient cooperative models [72]. Enhanced supply chain bargaining power makes it easier for firms to obtain trade credit support from upstream suppliers or partners in the form of extended payment terms or deferred settlements. This effectively reduces the cost of capital usage and strengthens firms’ ability and willingness to invest in real assets [71]. Consequently, greater access to commercial credit signifies stronger supply chain voice, higher firm status, and greater resource support within the alliance. The scale of commercial credit also reflects a firm’s ability to leverage strategic alliances to improve financing capabilities and construct trust-based mechanisms. As a critical financing tool, commercial credit improves corporate liquidity, enhances financial flexibility, and promotes supply chain coordination—thereby significantly reinforcing the impact of strategic alliances on corporate real investment. Based on the above analysis, the following hypotheses are proposed.

**Fig 1:**
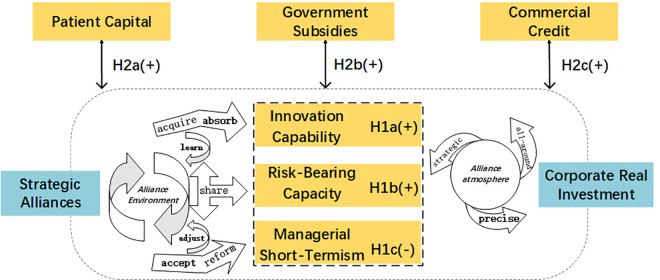
Theoretical model diagram.

**Hypothesis 2a:** Patient capital strengthens the positive impact of strategic alliances on corporate real investment.
**Hypothesis 2b:** Government subsidies strengthen the positive impact of strategic alliances on corporate real investment.
**Hypothesis 2c:** Commercial credit strengthens the positive impact of strategic alliances on corporate real investment.The theoretical model is illustrated in Fig 1.

## 4. Research design

### 4.1. Sample selection and data sources

This study utilizes A-share companies listed on the Shanghai and Shenzhen Stock Exchanges from 2009 to 2022 as the research sample. The sample excludes financial firm, and applies 1% winsorization at both ends for continuous variables to mitigate the influence of outliers. Firm-level financial and corporate governance data are obtained from the China Stock Market & Accounting Research Database(CSMAR), while strategic alliance data are collected from the Wind database. Following established literature, strategic alliance events are identified by screening corporate announcements that contain keywords such as “strategic alliance” in their titles. For firms that do not disclose a specific year of alliance participation, we follow conventional practices by assuming that the alliance spans the years t, t+1, and t+2, during which the firm is considered to be engaged in a strategic alliance.

### 4.2. Variable definitions and measurements

#### 4.2.1. Dependent variable.

Corporate Real Investment (invest) [73,74] Following standard definitions in existing literature, real investment is measured as: (Cash paid for the acquisition and construction of fixed assets, intangible assets, and other long-term assets – Cash received from the disposal of fixed assets, intangible assets, and other long-term assets)/ Total Assets. This equation captures the extent to which a firm allocates resources toward real, long-term asset investments.

#### 4.2.2. Independent variable.

Strategic Alliances (alliance) [75]. Referring to existing research, we identify strategic alliances by collecting company announcements whose titles contain any of the following five keywords: “strategic alliance,” “alliance cooperation,” “strategic cooperation,” “corporate alliance,” or “corporate union,” so as to comprehensively capture announcements related to strategic alliances.. The effective period of each alliance is determined based on the disclosed cooperation duration; if no specific duration is provided, the alliance is assumed to remain valid for three years. Admittedly, the research method employed in this paper has limitations. Keyword searches can only identify alliances mentioned in the announcements, and may overlook cooperation relationships that are not explicitly labeled as “strategic alliances” or informal agreements.. Based on the compiled announcements, a binary dummy variable, Alliance, is constructed, where 1 indicates that the listed firm either established a strategic alliance with another firm during the given year or was still within the valid cooperation period of a previously formed alliance, and 0 indicates that the firm did not participate in any strategic alliance during that year.

#### 4.2.3. Mediating variables.

This study utilizes three mediating variables: innovation capability (patent), managerial short-termism (myopi), and risk-bearing capacity (risk).

Innovation capability (patent) is measured, following established practice [76,77,78], as the natural logarithm of (1+ the sum of invention patents, utility model patents, and design patents applied for by the firm). A higher value indicates stronger innovation capability.

Managerial short-termism (myopia), per prior studies [79], Based on an established set of 43 keywords in both Chinese and English that reflect managerial short-term orientation—including 10 seed keywords such as “within days,” “several months,” and 33 extended keywords such as “just as,” “at the time when,” “difficulty,” and “predicament”—we calculate the proportion of the total frequency of these short-term orientation keywords in the MD&A section of the annual report for each year relative to the total word count of the MD&A text. This proportion is then multiplied by 100 to obtain the management short-termism index. A higher value of this index indicates a greater degree of managerial short-termism. The 43 keywords are: within days, several months, within the year, as soon as possible, immediately, at once, opportunity, at the time of, pressure, challenge, within the day, several days, subsequently, instantly, imminent, at the latest, no later than, critical moment, coinciding with, as arrival approaches, on the eve of, at the time of, encountering, just as, at the time when, difficulty, predicament, severe test, dual pressure, inflationary pressure, upward pressure, should act as soon as possible, as early as possible, at an early date, at an early stage, during, timing, as arrival approaches, financial pressure, environmental pressure, numerous difficulties, financing pressure, repayment pressure.

Risk-bearing capacity (risk), again based on the literature [80], is measured by the industry-adjusted standard deviation of return on assets (ROA). A higher standard deviation suggests greater willingness to bear risk.

#### 4.2.4. Moderating variables.

The moderating variables in this study include: patient capital (equity), government subsidies (subsidy), and commercial credit (NTC).

Patient capital (equity), following extant work [81], is measured using the proportion of institutional investor ownership. A higher proportion indicates a greater presence of stable, long-term-oriented investors.

Government subsidies (subsidy) data, per previous studies [82], are extracted from the notes to the financial statements in the CSMAR database. The amount of government grants received by the firm in the current year is used to represent the level of subsidy support.

Commercial credit (NTC), following prior studies [71], is measured as the ratio of the sum of accounts payable, notes payable, and advances from customers to total assets. This reflects the firm’s reliance on trade credit as a financing mechanism.

#### 4.2.5. Ccontrol variables.

Based on existing literature, this paper controls for the following potentially influential variables:1. Financial control vari ables, including leverage ratio (Lev) [19], return on assets (ROA) [75], Tobin’s Q (TobinQ) [19], and firm growth (Growth) [75]; 2. Governance control variables, including ownership concentration (TOP5) [75], board independence (Indep) [75], and CEO duality (Dual) [19]; 3. Firm characteristic control variables, including firm size (Size) [75], fixed asset ratio (FIXED) [19], and years listed (Age) [75]. In addition, the paper includes industry and time fixed effects, with specific variables shown in Table 1. Although this study mitigates confounding factors through control variables and fixed effects, endogeneity issues should still be carefully considered. Therefore, further tests for endogeneity will be conducted in the endogeneity testing section of the paper.

**Table 1:** Variable Definitions.

Variable Name	Symbol	Definition
Strategic alliance	alliance	Dummy variable: 1 if the firm participates in a strategic alliance in year *t*, 0 otherwise
Real investment	invest	(Cash paid for acquisition of fixed/ intangible/ other long-term assets − cash received from their disposal)/ total assets
Firm size	size	Natural logarithm of the number of employees
Leverage ratio	lev	Total liabilities divided by total assets
Return on assets	ROA	Net profit divided by total assets
Tobin’s Q	TobinQ	(Market value of tradable shares + non-tradable shares × book value per share + liabilities)/ total assets
Growth rate	growth	Growth rate of operating revenue
Fixed asset ratio	fixed	Net value of fixed assets divided by total asset value
Ownership concentration	top5	Total shareholding of top five shareholders divided by total shares
Board independence	indep	Proportion of independent directors
CEO duality	dual	Dummy variable: 1 if CEO and board chair are the same person, 0 otherwise
Years listed	age	Natural logarithm of the number of years since the firm was listed

### 4.3. Model construction

To empirically examine the relationship between firms’ participation in strategic alliances and their level of real investment in the manufacturing sector, the following baseline regression model is constructed:


$${invest}_{i,t}=\beta_{0}+\beta_{1}{alliance}_{i,t}+{control}_{i,t}+{industry}_{i}+{year}_{i}+\varepsilon_{i}$$ (1)

Where i denotes the individual firm, *t* represents the year, and *invest*_*i*,*t*_ denotes the real investment level of firm *i* year *t*. *alliance*_*i*,*t*_ is a dummy variable indicating whether the firm engaged in a strategic alliance in year t (1 = yes, 0 = no).

This study adopts the two-step mediation analysis approach to examine the mechanisms through which strategic alliances influence corporate real investment. To investigate the relationship between corporate participation in strategic alliances and the mediating variables, the following mechanism testing model is constructed. The corresponding model is specified as Equation (2) [83]:


$${\text{Mechanism}}_{i,t}=\alpha_{0}+\alpha_{1}{alliance}_{i,t}+{control}_{i,t}+{firm}_{i}+{industry}_{i}+\varepsilon_{i}$$ (2)

Mechanism denotes the mediating variables used in the mechanism analysis, specifically including risk-bearing capacity, innovation capability, and managerial short-termism.

To mitigate the influence of unobservable factors associated with industry characteristics and time-specific shocks on the empirical results, the model includes industry fixed effects (*industry*_*i*_) and year fixed effects (*year*_*i*_). Table 1 shows the relevant variables used in this paper and their definitions.

## 5. Empirical results and analysis

### 5.1. Descriptive statistics

Table 2 presents the descriptive statistics of the key variables. The average value of *corporate real investment* (*invest*) is 0.0567, with a standard deviation of 0.0628, indicating considerable variation in investment expenditure across sample firms. The minimum value is -0.0153, while the maximum reaches 0.3122, and the median is 0.0369. These figures suggest a right-skewed distribution, where a subset of high-investment firms raises the overall mean—an observation consistent with the typically uneven distribution of investment activities in the real economy.

**Table 2:** Descriptive Statistics of Key Variables.

Variable	Count	Mean	SD	Min	p50	Max.
invest	31142	0.0567	0.0628	−0.0153	0.0369	0.3122
alliance	31142	0.2219	0.4155	0.0000	0.0000	1.0000
allibilateral	31142	0.0227	0.1490	0.0000	0.0000	1.0000
alliequity	31142	0.0573	0.2324	0.0000	0.0000	1.0000
size	31142	22.2884	1.2606	19.8620	22.1031	26.2096
lev	31142	0.4262	0.2022	0.0502	0.4204	0.8982
ROA	31142	0.0421	0.0638	−0.2315	0.0398	0.2234
TobinQ	31142	2.0605	1.3014	0.8496	1.6446	8.4668
growth	31142	0.1690	0.3928	−0.5693	0.1082	2.4993
fixed	31142	0.2131	0.1581	0.0020	0.1810	0.6911
top5	31142	0.5299	0.1521	0.2003	0.5290	0.8851
indep	31142	0.3758	0.0535	0.3333	0.3636	0.5714
dual	31142	0.2762	0.4471	0.0000	0.0000	1.0000
age	31142	2.1556	0.8024	0.0000	2.3026	3.3322

Regarding the strategic alliance variables, the mean of *alliance* is 0.2219, indicating that approximately 22.2% of the firms in the sample engaged in strategic alliances. The means for *allibilateral* (bilateral contractual alliances) and *alliequity* (equity-based alliances) are 0.0227 and 0.0573, respectively, suggesting that only a small proportion of firms participated in these more specific alliance forms. Overall, the participation of firms in strategic alliances is notably unbalanced, providing a practical basis for further analysis on how different alliance types affect corporate investment behavior.

### 5.2. Baseline regression results

Table 3 presents the regression results regarding the impact of strategic alliances on corporate real investment. Column (1) reports the baseline regression with the full sample, where the coefficient of the independent variable *alliance* is significantly positive at the 1% level, indicating a robust positive relationship between strategic alliance participation and real investment. Column (2) focuses on bilateral contractual strategic alliances, where the coefficient of *alliance* is statistically insignificant, suggesting no clear effect on real investment. Column (3) shows results for equity-based strategic alliances, and indicates that the *alliance* coefficient is significantly positive at the 1% level. The above results indicate that participation in strategic alliances significantly enhances corporate entity investment, providing preliminary support for research hypothesis H1. Regarding economic interpretation, in Column (1), the coefficient of *alliance* suggests that, compared to firms not engaged in strategic alliances, those that do participate increase their real investment by approximately 12.3% (0.007/ 0.0567), relative to the sample mean of real investment. Furthermore, a one-standard-deviation increase in strategic alliance participation (0.4155) leads to an average increase of 5.13% in real investment (0.007 × 0.4155/ 0.0567). In Column (3), a one-standard-deviation increase in equity-based alliance participation (0.2324) corresponds to an average increase in real investment of approximately 2.5% (0.0061 × 0.2324/ 0.0567). These results confirm that strategic alliances significantly promote corporate real investment; in addition, the type of alliance matters, with equity-based alliances exerting a stronger influence than bilateral contractual alliances. Before conducting the regression analysis, both Industry fixed effects(Industry FE) and Year fixed effects(Year FE) were controlled for to mitigate potential omitted variable bias.

**Table 3:** Baseline Regression Results.

	(1)	(2)	(3)
Variable	invest	invest	invest
alliance	0.0070***		
	(6.6543)		
alliance × allibilateral		0.0023	
		(0.7457)	
alliance × alliequity			0.0061***
			(3.3526)
size	0.0053***	0.0056***	0.0055***
	(8.1956)	(8.6325)	(8.5233)
lev	0.0206***	0.0208***	0.0209***
	(6.6424)	(6.6894)	(6.7062)
ROA	0.1162***	0.1128***	0.1141***
	(15.1406)	(14.7224)	(14.8302)
TobinQ	0.0008**	0.0009**	0.0008**
	(1.9961)	(2.1844)	(2.1113)
growth	0.0235***	0.0237***	0.0237***
	(16.8732)	(17.0042)	(16.9406)
fixed	0.0960***	0.0951***	0.0954***
	(19.5634)	(19.3018)	(19.3784)
top5	−0.0074*	−0.0090**	−0.0086**
	(−1.9078)	(−2.3136)	(−2.2001)
indep	0.0040	0.0037	0.0039
	(0.4620)	(0.4264)	(0.4542)
dual	0.0062***	0.0063***	0.0063***
	(5.6525)	(5.7289)	(5.7136)
age	−0.0214***	−0.0216***	−0.0215***
	(−24.9615)	(−25.0444)	(−24.9702)
constant	−0.0547***	−0.0591***	−0.0584***
	(−4.1168)	(−4.4074)	(−4.3559)
Observations	31,141	31,141	31,141
Adjusted R-squared	0.2447	0.2428	0.2433
Industry FE	YES	YES	YES
Year FE	YES	YES	YES

**Note:** Significance levels: *** p < .01, ** p < .05, * p < .10. The same notation applies in all subsequent tables.

### 5.3. Mechanism testing

Baseline regression results indicate that firms’ participation in strategic alliances contributes significantly to promoting corporate real investment. However, the specific pathways through which this effect is realized remain to be empirically validated.

As discussed in the theoretical analysis, strategic alliances can operate through multiple channels: by reducing agency costs and operational expenses, by enhancing managerial risk tolerance, or by eliminating the “island effect” often observed in innovation processes. Moreover, alliances help mitigate the uncertainty risks that individual firms face during innovation. Together, these mechanisms contribute to lowering both internal and external investment risks, thereby strengthening firms’ capacity and willingness to undertake real investment. Therefore, this study adopts an integrated perspective from both internal and external dimensions of the firm to construct a logical framework and a system of simultaneous equations, aiming to examine the mechanisms through which strategic alliances influence corporate real investment. Specifically, it investigates how strategic alliances affect real investment via three mediating channels: managerial short-termism, corporate innovation capability, and risk-bearing capacity.

Table 4 presents the results of the mechanism analysis examining the pathways through which strategic alliances influence corporate real investment. The regression coefficient in Column 1 for corporate strategic alliances (Alliance) on corporate innovation capability (patent) is significantly positive at the 1% level, indicating that strategic alliances enhance the firm’s innovation capability. These results suggest that corporate involvement in strategic alliances boosts innovation capability, which in turn promotes real investment activities, supporting the research hypothesis 1a of this study.The regression coefficient in Column 2 for corporate strategic alliances (Alliance) on corporate risk-bearing capacity (risk) is significantly positive at the 1% level, indicating that strategic alliances increase the firm’s risk-bearing capacity. These results suggest that corporate involvement in strategic alliances enhances risk-bearing capacity, which in turn promotes real investment activities, supporting the research hypothesis 1b of this study.The regression coefficient in Column 3 for corporate strategic alliances (Alliance) on managerial short-termism (myopia) is significantly negative at the 1% level, indicating that strategic alliances mitigate managerial short-termism. These results suggest that corporate participation in strategic alliances curbs managerial myopia, thereby promoting real investment activities and supporting the research hypothesis 1c of this study.

**Table 4:** Mechanism Test Results.

	(1)	(2)	(3)
Variable	Patent	risk	myopia
alliance	0.0970***	0.0023***	−0.0110***
	(3.5454)	(3.0880)	(−6.7593)
size	0.6540***	−0.0031***	0.0020*
	(35.8148)	(−7.3816)	(1.8828)
lev	−0.0406	−0.0059**	0.0259***
	(−0.4404)	(−2.2227)	(4.9494)
ROA	1.0571***	−0.2150***	−0.0757***
	(4.9411)	(−22.7598)	(−5.9471)
TobinQ	0.0248**	0.0045***	−0.0019**
	(2.2007)	(12.0540)	(−2.5000)
growth	−0.0831***	0.0075***	−0.0116***
	(−3.6865)	(8.2727)	(−8.4753)
fixed	−0.4896***	−0.0110***	0.0293***
	(−3.9641)	(−3.9442)	(4.3985)
top5	−0.1368	−0.0006	−0.0020
	(−1.1545)	(−0.2142)	(−0.2867)
indep	−0.3678	0.0091	0.0073
	(−1.3582)	(1.4744)	(0.4364)
dual	0.0432	0.0010	−0.0070***
	(1.4614)	(1.2966)	(−4.0335)
age	−0.0531**	0.0022***	0.0131***
	(−2.2758)	(3.5907)	(10.1187)
Constant	−11.5651***	0.0958***	0.0396*
	(−29.1295)	(11.2490)	(1.6461)
Observations	31,141	31,141	31,141
Adjusted R-squared	0.4887	0.1744	0.1727
Industry FE	YES	YES	YES
Year FE	YES	YES	YES

### 5.4. Moderation effect test

The impact of strategic alliances on corporate real investment may vary depending on firms’ capital acquisition capability. As a form of external cooperation, strategic alliances enable firms to better identify and seize investment opportunities; however, the realization of these opportunities often relies heavily on external financial support. A favorable external financing environment can ease firms’ capital constraints and stimulate their investment decisions [84,85]. The cooperative relationships established within alliances foster positive resource synergy effects, which enhance firms’ financing performance, reduce investment costs and risks, and ultimately increase the likelihood that alliance-generated opportunities will translate into actual investment behaviors [86].

This, this study selects patient capital, government subsidies, and commercial credit as moderating variables in the relationship between strategic alliances and corporate real investment. Patient capital helps to alleviate short-term performance pressure, thereby supporting long-term investment. Commercial credit reduces financing costs and expands funding sources, facilitating firms’ access to capital needed for investment. Government subsidies provide direct financial support, reducing investment risk and easing financial burdens. Together, these three factors constitute a favorable external financing environment that amplifies the investment-promoting effect of strategic alliances. Table 5 presents the regression results testing the moderating effects of patient capital, government subsidies, and commercial credit on the relationship between strategic alliances and corporate real investment. Column (1) reports the result including the interaction term *alliance* × *equity* (patient capital), Column (2) includes *alliance* × *LNSubsidy* (government subsidies), and Column (3) includes *alliance* × NTC (commercial credit). In all three columns, the interaction terms are positively significant at least at the 10% level, indicating that firms with stronger capital acquisition capability benefit more from strategic alliances in terms of real investment enhancement. These findings suggest that favorable capital access conditions—whether in the form of long-term institutional ownership, external financial support, or trade credit—strengthen the positive effect of strategic alliances on corporate real investment by facilitating the transformation of alliance-driven opportunities into productive investment decisions. At the same time, the above proves that research hypothesis 2a, hypothesis 2b, and hypothesis 2c are established.

**Table 5:** Moderation Test Results.

	(1)	(2)	(3)
Variables	invest	invest	invest
alliance × equity	0.0033**		
	(2.0225)		
alliance × LNSubsidy		0.0033*	
		(1.7558)	
alliance × NTC			0.0037**
			(1.9669)
size	0.0054***	0.0046***	0.0051***
	(8.4860)	(6.6304)	(8.0554)
lev	0.0207***	0.0202***	0.0191***
	(6.6803)	(6.4984)	(6.1068)
ROA	0.1168***	0.1150***	0.1150***
	(15.2477)	(14.9791)	(14.9399)
TobinQ	0.0008**	0.0007*	0.0008**
	(2.1567)	(1.9207)	(2.0026)
growth	0.0234***	0.0237***	0.0236***
	(16.8227)	(16.9575)	(16.9438)
fixed	0.0964***	0.0956***	0.0947***
	(19.6496)	(19.4418)	(19.3253)
top5	−0.0070*	−0.0070*	−0.0080**
	(−1.7933)	(−1.8076)	(−2.0738)
indep	0.0034	0.0046	0.0039
	(0.3963)	(0.5395)	(0.4594)
dual	0.0061***	0.0062***	0.0062***
	(5.5856)	(5.6478)	(5.6591)
age	−0.0211***	−0.0214***	−0.0215***
	(−24.4802)	(−24.9189)	(−24.9644)
Constant	−0.0577***	−0.0419***	−0.0518***
	(−4.3446)	(−2.9189)	(−3.9121)
Observations	31,141	31,141	31,141
Adjusted R-squared	0.2452	0.2451	0.2453
Industry FE	YES	YES	YES
Year FE	YES	YES	YES

### 5.5. Robustness tests

To ensure the reliability of the empirical findings, several robustness are conducted, The results are shown in Table 6.

**Table 6:** Robustness Test Results.

	(1)	(2)	(3)	(4)
Variable	invest2	invest	invest	invest
alliance	0.0076***		0.0067***	0.0077***
	(5.2056)		(6.3130)	(5.8871)
calliance		0.0061***		
		(6.3287)		
size	0.0056***	0.0052***	0.0050***	0.0046***
	(7.6692)	(8.0842)	(7.7099)	(5.6919)
lev	0.0055	0.0206***	0.0212***	0.0170***
	(1.2649)	(6.6513)	(6.7606)	(4.4692)
ROA	0.0088	0.1167***	0.1187***	0.1339***
	(0.7657)	(15.1833)	(15.0900)	(13.2064)
TobinQ	−0.0011**	0.0008**	0.0007*	−0.0004
	(−2.4097)	(1.9695)	(1.7886)	(−0.9309)
growth	0.0554***	0.0235***	0.0234***	0.0232***
	(14.0543)	(16.8647)	(16.6609)	(14.0437)
fixed	0.0201***	0.0961***	0.0968***	0.0946***
	(3.3995)	(19.6075)	(19.2700)	(16.1630)
top5	0.0016	−0.0071*	−0.0079**	−0.0028
	(0.3348)	(−1.8216)	(−2.0117)	(−0.5859)
indep	0.0012	0.0041	0.0055	0.0027
	(0.1134)	(0.4747)	(0.6354)	(0.2550)
dual	0.0072***	0.0062***	0.0061***	0.0068***
	(5.2906)	(5.6505)	(5.5449)	(4.9376)
age	−0.0117***	−0.0214***	−0.0213***	−0.0201***
	(−11.2042)	(−24.9254)	(−24.5897)	(−18.1188)
Constant	−0.0731***	−0.0536***	−0.0502***	−0.0419**
	(−4.8045)	(−4.0280)	(−3.7400)	(−2.5264)
Observations	31,141	31,141	31,064	20,484
Adjusted R-squared	0.1853	0.2448	0.2533	0.2386
Industry FE	YES	YES	YES	YES
Year FE	YES	YES	YES	YES

*Alternative Measurements of Key Variables*: To address potential biases from relying on a single measurement, we replace the independent variable with the number of strategic alliances (*calliance*) [87] and redefine the dependent variable as an alternative measure of real investment (*invest2*). This alternative measure is calculated by adding the cash paid for the construction of fixed assets, intangible assets, and other long-term assets to the net cash paid for acquiring subsidiaries and other business units, and then scaling the total by the firm’s beginning-of-period total assets. This metric also captures the firm’s long-term capital expenditures in both physical and strategic assets. The regression results, as reported in Table 6, Columns (1) and (2), show that both *alliance* and *calliance* are significantly positive at the 1% level, indicating a strong and consistent positive relationship between strategic alliances and corporate real investment. These results indicate that the positive effect of strategic alliances on corporate real investment remains robust, thereby confirming the reliability and consistency of the baseline regression findings.

*Time–Industry Interaction Fixed Effects*: To control for and eliminate potential biases, and to ensure the reliability and robustness of the regression results, this study incorporates industry–year interaction fixed effects in addition to the standard year and industry fixed effects, and re-estimates the regression model accordingly. The regression results, as shown in Table 6, Column (3), are consistent with those of the baseline model, further confirming the robustness of the main findings.

*Sample Period Adjustment*: The outbreak of COVID-19 in December 2019 significantly increased uncertainty in the external environment and disrupted supply and demand conditions, which may have had a substantial impact on firms’ real investment decisions. To avoid the potential distortions caused by the COVID-19 pandemic on firms’ real investment, this study excludes sample data from 2020 to 2022 and reexamines the impact of strategic alliances on corporate real investment. The regression results, shown in Table 6, Column (4), indicate that the coefficient on *alliance* remains significantly positive, consistent with the baseline regression results.

### 5.6. Endogeneity test

*Instrumental Variable Approach*: To address potential endogeneity concerns, this study employs an instrumental variable (IV) method, following the approach of Chou et al.[52] To do so, a lagged proportion of other listed firms in the same industry (excluding the focal firm) that participated in strategic alliances are selected as the instrumental variable, denoted as *alliance*_*iv*. Table 7, Columns (1) and (2), present the two-stage regression results using the IV approach. The results show that the instrument (*alliance_iv*) successfully passes both the under-identification test and the weak instrument test, indicating its validity and relevance. Table 7, Column (2), shows that the coefficient of *alliance* remains significantly positive at the 1% level, indicating that after addressing potential endogeneity, the core finding—that strategic alliances promote corporate real investment—holds.

**Table 7:** Endogeneity Test Results.

	(1)	(2)	(3)	(4)	(5)
Variable	alliance	invest	invest	f.invest	invest
alliance_iv	0.9873***				
	(26.1633)				
alliance		0.0183***	0.0027**	0.0048***	0.0056***
		(3.4967)	(2.5200)	(4.1367)	(4.3176)
size	0.0457***	0.0047***	0.0111***	0.0030***	0.0061***
	(9.5863)	(6.9249)	(8.0390)	(4.0867)	(6.7699)
lev	0.0363	0.0202***	0.0149***	0.0146***	0.0213***
	(1.4518)	(6.5067)	(3.0283)	(4.0939)	(4.8840)
ROA	−0.5035***	0.1218***	0.0647***	0.1380***	0.1176***
	(−8.3504)	(15.0092)	(8.3554)	(14.2164)	(11.1576)
TobinQ	0.0090***	0.0006*	0.0007*	0.0034***	0.0016***
	(2.9309)	(1.6455)	(1.6488)	(6.9795)	(2.7773)
growth	0.0244***	0.0232***	0.0180***	0.0082***	0.0196***
	(3.6599)	(16.5335)	(13.6201)	(7.3825)	(9.3727)
fixed	−0.1307***	0.0976***	−0.0562***	0.0831***	0.1079***
	(−4.2895)	(19.6899)	(−6.5007)	(15.1394)	(15.6336)
top5	−0.2303***	−0.0047	0.0346***	−0.0113***	−0.0091*
	(−7.7544)	(−1.1629)	(4.5041)	(−2.6258)	(−1.6722)
indep	−0.0431	0.0045	−0.0035	0.0026	−0.0032
	(−0.6256)	(0.5211)	(−0.3465)	(0.2692)	(−0.2597)
dual	0.0171**	0.0060***	0.0039***	0.0060***	0.0079***
	(2.0311)	(5.4637)	(2.9560)	(4.7678)	(5.0002)
age	−0.0188***	−0.0212***	−0.0233***	−0.0179***	−0.0214***
	(−3.1723)	(−24.5794)	(−10.5314)	(−18.8449)	(−15.9055)
Constant	−0.8318***		−0.1601***	−0.0107	−0.0747***
	(−8.3111)		(−5.1988)	(−0.7026)	(−3.9938)
Observations	31,141	31,141	30,672	23,660	11,165
Adjusted R-squared	0.1166	0.1456	0.4536	0.2038	0.2482
Industry FE	YES	YES	YES	YES	YES
Year FE	YES	YES	YES	YES	YES

*Firm Fixed Effects Test*: The firm fixed effects test is conducted to control for unobserved, time-invariant characteristics specific to each firm that may bias the estimation results. By including firm fixed effects, this approach helps to mitigate potential endogeneity issues arising from omitted variables or self-selection bias. The regression results, shown in Table 7, Column (3), indicate that the coefficient on *alliance* remains significantly positive, suggesting that the main conclusion is robust even after accounting for firm-level heterogeneity.

*Accounting for Dynamic Timing Consistency*: Since strategic alliance data in this study are obtained from corporate announcements, the actual commencement of alliance cooperation may lag behind the announcement date. To address this potential timing discrepancy—and to further mitigate reverse causality concerns—we re-estimate the model using the lead of the dependent variable (*f.invest*), which shifts real investment forward by one period. As shown in Table 7, Column (4), the coefficient on *alliance* remains significantly positive, indicating that the main conclusion holds even after accounting for dynamic timing consistency.

*Propensity Score Matching*: Firms may reduce their willingness to engage in real investment due to operational challenges or drastic changes in the external environment. At the same time, given that only a limited number of firms participate in strategic alliances, the uneven sample distribution may potentially bias the regression results. To address potential sample selection bias and distributional imbalance, this study employs the nearest-neighbor matching method, using control variables as matching covariates and adopting a 1:1 matching ratio between treated and control firms. After passing the covariate balance test, the model is re-estimated based on the matched sample. As shown in Table 7, Column (5), the regression results remain consistent with the baseline findings, confirming the reliability and robustness of the study’s conclusions.

## 6. Conclusion and policy implications

This study incorporates strategic alliances and corporate real investment into a unified research framework to examine the direction, mechanisms, and magnitude of the impact of strategic alliances on real investment. The findings reveal that strategic alliances enhance firms’ risk-bearing capacity, suppress managerial short-termism, and strengthen innovation capability, thereby promoting real investment. Further analysis shows that the positive effect of strategic alliances becomes significantly stronger when firms have greater capital acquisition capability. Moreover, the findings show that equity-based strategic alliances exert a stronger influence on real investment compared to bilateral contractual alliances, highlighting the importance of alliance structure in shaping investment outcomes.

By empirically examining the relationship and underlying mechanisms between strategic alliances and corporate real investment, this study enriches the existing literature on both the economic consequences of strategic alliances and the determinants of real investment. At the same time, this study offers practical insights for regulators, corporate management, and stakeholders on how to strengthen strategic alliance relationships, enhance firms’ resource allocation efficiency, and improve risk supervision and response mechanisms. First, regarding regulatory authorities, it is necessary to promote the development of patient capital and fiscal incentive mechanisms by formulating policy systems that support long-term corporate investment, and to encourage firms to participate in high-quality strategic alliances. On this basis, the requirements for resource integration and equity ratio in alliance cooperation can be further clarified, guiding enterprises to establish stable and long-term oriented alliance relationships. In addition, measures such as tax reductions and R&D subsidies should be implemented to strengthen support for corporate innovation, thereby enhancing their willingness and capability to invest in the real economy. Second, for corporate management, a long-term strategic orientation should be established, and alliance cooperation should be incorporated into the company’s investment planning to avoid strategic deviations caused by short-term behaviors. In alliance practice, managers should clarify capital investment and resource commitment, optimize governance structures, and promote resource sharing and risk sharing. At the same time, efforts should be made to improve corporate credit ratings, expand cooperation channels with financial institutions and investors, and enhance financing capabilities to ensure the smooth advancement of alliance investments. Finally, with respect to stakeholders, active support should be given to companies in creating long-term value through strategic alliances. Especially in emerging markets, attention should be paid to the role of equity-based strategic alliances in capital support, resource integration, and capability enhancement, thereby promoting enterprises to build robust financing structures and avoid the financialization dilemma of “divorcing from the real economy” driven by short-term profits. This will help realize sustainable long-term investment growth.

This study provides an empirical analysis of the impact of strategic alliances on corporate investment, but it still has certain limitations. First, the research only analyzes publicly listed companies in China. Future studies could consider expanding the sample to include firms from more countries and regions to verify the generalizability of the results. Second, this study uses the same regression model as previous research. Other researchers may attempt to use alternative nonlinear models or different research methodologies to further explore the economic consequences of strategic alliances. Finally, this study relies on publicly available financial statements and company annual reports as the primary data sources. While these data sources are generally reliable, they do not fully reflect non-financial factors that could significantly influence corporate investment decisions. Future research could adopt different data processing methods to address data with uncertainty and ambiguity, enriching the article’s findings.

## Supporting information

S1 Data Data materials. (XLSX)
